# A Novel Complotype Combination Associates with Age-Related Macular Degeneration and High Complement Activation Levels *in vivo*

**DOI:** 10.1038/srep26568

**Published:** 2016-05-31

**Authors:** Constantin C. Paun, Yara T. E. Lechanteur, Joannes M. M. Groenewoud, Lebriz Altay, Tina Schick, Mohamed R. Daha, Sascha Fauser, Carel B. Hoyng, Anneke I. den Hollander, Eiko K. de Jong

**Affiliations:** 1 Radboud university medical center, Department of Ophthalmology, Nijmegen, The Netherlands; 2 Radboud university medical center, Donders Institute for Brain, Cognition and Behaviour, Nijmegen, The Netherlands; 3 Radboud university medical center, Department for Health Evidence, Nijmegen, The Netherlands; 4 University Hospital of Cologne, Department of Ophthalmology, Cologne, Germany; 5 Leiden University Medical Center, Department of Nephrology, Leiden, The Netherlands; 6 Radboud university medical center, Department of Human Genetics, Nijmegen, The Netherlands

## Abstract

The complement system is the first line of defense against foreign intruders, and deregulation of this system has been described in multiple diseases. In age-related macular degeneration (AMD), patients have higher complement activation levels compared to controls. Recently, a combination of three single nucleotide polymorphisms (SNPs) in genes of the complement system, referred to as a complotype, has been described to increase complement activation *in vitro*. Here we describe a novel complotype composed of *CFB* (rs4151667)-*CFB* (rs641153)-*CFH* (rs800292), which is strongly associated with both AMD disease status (p = 5.84*10^−13^) and complement activation levels *in vivo* (p = 8.31*10^−9^). The most frequent genotype combination of this complotype was associated with the highest complement activation levels in both patients and controls. These findings are relevant in the context of complement-lowering treatments for AMD that are currently under development. Patients with a genetic predisposition to higher complement activation levels will potentially benefit the most of such treatments.

The complement system is part of our innate immunity where it acts as a first line of defense against foreign intruders^1^ and as a surveillance system to discriminate between healthy host tissue, cellular debris and apoptotic cells^2^. The complement system can be triggered through one of its three pathways: the classical pathway (CP), the lectin pathway (LP) and the alternative pathway (AP). All three pathways converge at the level of complement component 3 (C3), which is cleaved into C3a (a potent proinflammatory molecule) and C3b (an opsonin)^1^.

After C3 cleavage, a subsequent cascade of enzymatic reactions lead to the formation of the membrane-attack-complex, which is responsible for disrupting the target cell membrane by forming transmembrane pores^3^. Unlike the CP and the LP, which need certain triggers to become activated, the AP is always at a low level of activation via a process called “tick-over”^4^, a spontaneous conversion of C3 to a bioactive form C3(H_2_O)^5^. This conversion leads to a conformational change that allows for the binding of complement factor B (FB), similar to C3b^5^ and, through a series of subsequent steps, forms the initial C3 convertase C3(H_2_O)Bb^1^. This convertase cleaves C3 molecules into C3a and C3b^5^,^6^. In plasma, AP amplification is controlled by complement factor H (FH), which inactivates the C3 convertase and catalyses complement factor I (FI) degradation of C3b^7^. Dysregulation of the AP is associated with a number of diseases^8^, a strong example being age-related macular degeneration (AMD)^9^,^10^,^11^,^12^.

AMD is a progressive retinal disease that leads to vision loss in the elderly population^13^. It is a multifactorial disease caused by both genetic and environmental factors. Several lines of evidence support the involvement of the complement system in the pathology of AMD. Some of the highest genetic risk for AMD is conferred by single nucleotide polymorphisms (SNPs) in or near genes of the complement system^14^. Additionally, complement activation levels in plasma/serum are significantly higher in patients compared to controls^9^,^10^,^11^,^12^, and complement components have been described in the composition of retinal deposits called drusen, which are a hallmark of the disease^15^.

Currently, AMD therapies that aim to inhibit or lower complement activation are being developed^16^,^17^, but it has been suggested that one of these inhibitors, lampalizumab, is effective only in a subset of patients that carry a specific genotype^18^. Therefore, it is important to understand the genetic risk factors that influence complement activation in order to identify those individuals that would benefit the most from such treatments.

Several studies have evaluated the effect of SNPs on complement activity, and only moderate effects have been observed^19^,^20^,^21^. *In vitro* studies show that complement activity can increase six-fold when multiple SNPs in the complement system interact together^20^. Such combinations of SNPs in the complement system are called complotypes. Harris *et al.* defined the complotype as any inherited pattern of genetic variants in complement genes which alters risk for both inflammatory disorders and infectious diseases involving the complement system^22^. Until now, the best studied complotype *in vitro* is composed of three functional variants from the AP: *C3* (rs2230199 p.R102G), *CFB* (rs641153 p.R32Q) and *CFH* (rs800292 p.V62I). All three SNPs are individually associated with AMD^23^,^24^,^25^. Although the presence of all three SNPs led to markedly higher complement activity *in vitro*, the effect of the complotype has so far neither been investigated in human plasma samples, representative of the *in vivo* situation, nor been associated with any disease.

In a recent study, we have found another functional SNP in *CFB* (rs4151667) to be more strongly associated with complement activation^9^ than the individual SNPs in the most studied complotype (*C3* (rs2230199), *CFB* (rs641153) and *CFH* (rs800292)). The aims of this study, therefore, are: 1) to expand the complotype with the *CFB* variant (rs4151667) we found to be highly associated with complement activity; 2) to evaluate the relation of the complotype with complement activation in human plasma samples, representative of the *in vivo* situation; and 3) to investigate the association between the complotype and AMD.

## Results

The study was performed in three consecutive steps. First, the individual associations of *CFH* (rs800292), *CFB* (rs4151667), *CFB* (rs641153) and *C3* (rs2230199) with AMD and with complement activation were verified. Next, we determined the most informative complotype for complement activation. Finally, we analyzed the association of the resulting complotype with the disease and with complement activation.

### Individual association of *CFH* (rs800292), *CFB* (rs4151667), *CFB* (rs641153) and *C3* (rs2230199) with AMD and complement activation

In a previous study, *CFH* rs800292, *CFB* rs4151667, *CFB* rs641153 and *C3* rs2230199 were tested for their association with AMD in 2,655 individuals^9^. For the purpose of this study, 387 additional individuals were genotyped, amounting to a total of 3,042 subjects (1,615 AMD and 1,427 Controls). The mean age was 75 for AMD and 70 for controls. The gender distribution was: 41% males to 59% females. All four SNPs were significantly associated with AMD (Supplementary Table 1). SNPs *CFH* rs800292 (minor allele A), *CFB* rs4151667 (minor allele A) and *CFB* rs641153 (minor allele A) are protective, whereas the *C3* rs2230199 (minor allele C) infers increased risk of AMD.

**Figure 1:**
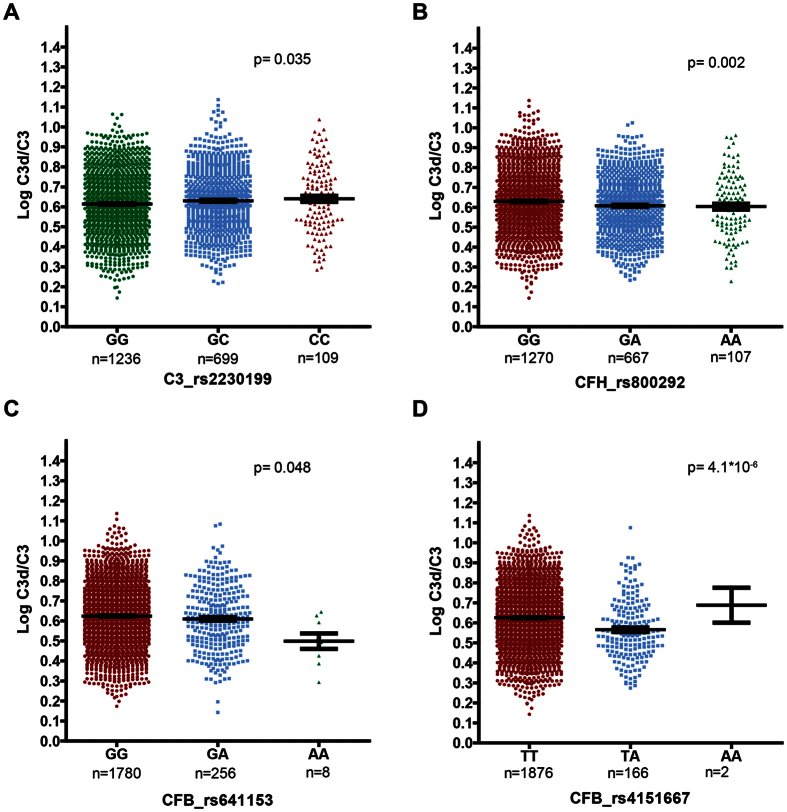
Plasma complement activation levels (log-transformed C3d/C3 ratio) for *C3*, *CFH* and *CFB* genotype groups. Each genotype per SNP is plotted on the X axis in an individual dot plot. The homozygous genotypes conferring increased risk for AMD are indicated in red; the homozygous genotypes that are protective for AMD are indicated in green. The number of individuals carrying a specific genotype is indicated below each genotype. The Y axis represents the Log-transformed C3d/C3 ratio level as a measure of complement activation. The p-values represent the overall significance for each SNP included in the model.

To determine the association of these SNPs with complement activation levels, *CFH* (rs800292), *CFB* (rs4151667), *CFB* (rs641153) and *C3* (rs2230199) were included in a single general linear model, corrected for age, gender, body mass index (BMI) and disease status. The model revealed significant independent associations with complement activation levels for all four SNPs. Figure 1 illustrates P-values, mean log-transformed complement activation levels and genotype distribution for the four tested SNPs.

When we looked at the difference in mean complement activation level between the genotypes for each SNP, the high-risk *C3* (rs2230199) genotype (GG) showed higher complement activation levels than the heterozygous (CG) and ancestral (CC) genotype. The protective *CFH* (rs800292) genotype (AA) showed lower complement activation levels than the other genotypes (Fig. 1). However, a statistically significant difference in mean complement levels was only observed between the heterozygous genotype (GA) and the major genotype (GG) (p = 0.002), presumably due to the limited number of individuals carrying the AA genotype. The protective *CFB* (rs641153) genotype (AA) and the heterozygous (GA) genotype displayed lower mean complement activation levels than the ancestral (GG) genotype. For *CFB* (rs4151667), the homozygous protective genotype for AMD (AA) could not be statistically compared to the homozygous ancestral genotype (TT), due to low number of individuals in this genotype group. The observed effects are driven by the difference in mean complement activation levels between the heterozygous (TA) genotype and the ancestral (TT) genotype (Fig. 1).

### The most informative SNP combination in determining complement activation or AMD status

As all four SNPs were individually and independently associated with both complement activation and AMD status, the next step aimed to assess which combination of SNPs best predicted these associations. It was impossible to introduce genotype combinations of all 4 SNPs into the model because of the very low samples number of individuals in each of the resulting groups. For this reason, only combinations of 3 SNPs were considered.

In order to determine which combination of SNPs could best explain complement activation and disease status, two random forest analyses were performed. In the first analysis, the ratio of C3d/C3 as a measure of complement activation was used as the dependent variable, whereas the second analysis was classified on AMD disease status. Variable importance analyses in both tests revealed that the SNP combination composed of *CFB* (rs4151667)-*CFB* (rs641153)-*CFH* (rs800292) was the strongest predictor for both complement activation and AMD status (Table 1). For the purpose of clarity, this combination of SNPs will be referred to as the novel complotype in the remainder of the manuscript.

### Association of the novel complotype with AMD

Mathematically, there are 27 possible genotype combinations for a complotype composed of three SNPs. To ensure a meaningful interpretation of the statistical analyses, we included only those genotype combinations that were represented by at least ten individuals in both the patients and controls group. In our cohort, we observed seven genotype combinations that met these criteria. The distribution of all genotype combinations in our cohort is shown in Supplementary Table 2.

To determine the association of the novel complotype with AMD, a logistic regression analysis was performed. A strong overall association of the novel complotype with AMD (p = 5.84*10^−13^) was observed. In our analysis of the genotype combinations within the novel complotype, the most frequent genotype combination found in controls (TT-GG-GG) was set as reference. The logistic regression analyses corrected for age and gender revealed that, in comparison to TT-GG-GG, the other six genotype combinations were protective for AMD (Table 2).

### Association of the novel complotype with complement activation

**Figure 2:**
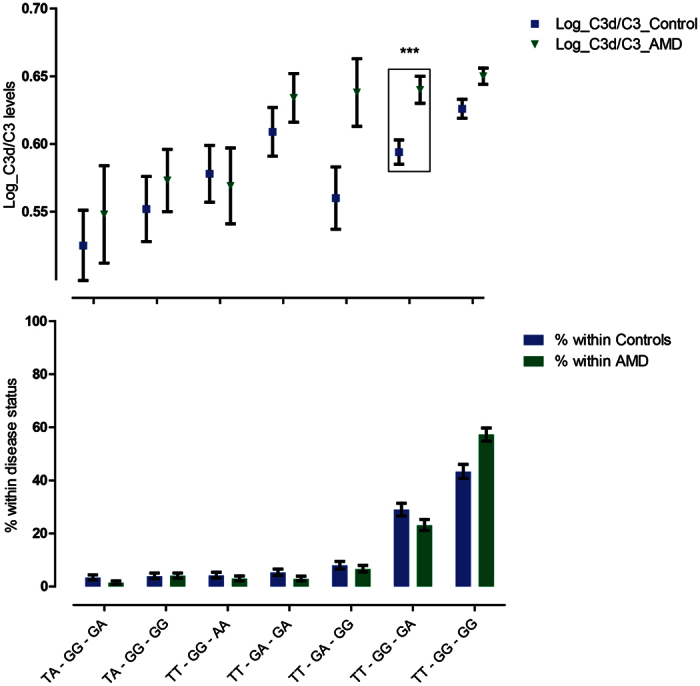
Mean C3d/C3 level and frequency of genotype combinations in AMD patients and controls. The blue and green bars represent the percentage of individuals carrying a specific genotype combination within their own disease status. The green triangles and blue squares represent mean C3d/C3 values for the corresponding genotype combination. The only genotype combination showing a significant difference in complement levels between AMD patients and controls was observed for TT-GG-GA with a p-value of 3*10^−4^ (after Bonferroni correction statistical significance is achieved at p < 0.007).

Finally, to determine the association of the novel complotype with complement activation, a general linear model was built, corrected for age, gender, BMI and disease status. This model showed that the novel complotype was highly associated with complement activation levels (p = 8.31*10^−9^). When we compared the different genotype combinations with one another, the TT-GG-GG combination was associated with the highest mean complement activation levels (Fig. 2). The difference in mean complement activation levels between all genotype combinations, tested in a post-hoc Bonferroni corrected manner, are presented in Supplementary Table 3. When comparing complement activation levels between AMD patients and controls, we only observed a significant difference for genotype combination TT-GG-GA (Fig. 2).

## Discussion

In a large case-control study, we show that carrying multiple AMD protective genotypes for *CFB* (rs4151667)*, CFB* (rs641153) and *CFH* (rs800292) leads to lower levels of complement activation in plasma compared to the most frequent genotype combination of these SNPs in control individuals. This novel complotype was identified as the most predictive SNP combination for determining both complement activation levels and AMD status. This combination of SNPs, drawn from an *in vivo* setting, is different from what has previously been suggested on the basis of *in vitro* data^20^.

It is well established that SNPs in complement components *C3*, *CFB* and *CFH* influence the risk for AMD^24^,^25^,^26^. In this study, we confirmed that four common functional SNPs, *CFH* (rs800292), *CFB* (rs4151667), *CFB* (rs641153) and *C3* (rs2230199) are associated with AMD. The minor alleles of the *CFH* and the *CFB* SNPs are protective^23^,^24^, whereas the minor allele of the *C3* SNP confers increased risk of AMD^25^. The well-known AMD SNP *CFH* (rs1061170; Tyr402His) was not included in this study because it was not associated with complement activation in our previous study^9^. This SNP was not described to alter AP convertase regulation, but rather it displays differential binding to C-reactive protein^27^ and malondialdehyde^28^.

Higher levels of systemic complement activation in patients compared to controls have been described in multiple studies^9^,^10^,^12^,^29^. As the proteins encoded by *CFH*, *CFB* and *C3* are key components of the AP of the complement system^30^, the contribution of these SNPs to disease susceptibility possibly comes from their impact on AP activation.

FH is a major regulator of the AP^31^. One of the ways in which it down-regulates complement activity is to bind C3b as a cofactor for its inactivation^32^. The A allele (p.62I) of the *CFH* (rs800292, pV62I) SNP is a gain of function variant. *In vitro* experiments showed that the resulting protein binds more efficiently to C3b than the protein resulting from the G allele (p.V62) of this SNP^19^, thus leading to more complement inhibition. This is in line with our results, demonstrating that the *CFH* (rs800292) GG genotype was associated with decreased risk for AMD and lower levels of complement activation than the AA genotype.

FB binds hydrolyzed C3(H_2_O) or C3b, which is then cleaved by complement factor D to form the AP C3 convertase that cleaves C3 to C3a and C3b^22^, thus fueling the AP amplification loop. The A (p.32Q) allele of rs641153 (p.R32Q) leads to a FB protein with decreased potential to form the C3 convertase and amplify complement activation^33^. The second *CFB* SNP (rs4151667) (p.L9H) resides in the signal peptide, and it has been proposed that it could alter CFB secretion^24^. In this study, the A alleles of both *CFB* SNPs were found to be protective for AMD and to lead to lower complement activation levels, even in heterozygous state, than the major homozygous genotype. The homozygous protective genotypes for CFB (rs4151667) were too rare for any reliable conclusions to be drawn.

C3 plays a central role in the complement system^34^. The G (p.102G) allele of *C3* (rs2230199, p.R102G) decreases the efficiency of regulation of C3b by FH, thus leading to an increase in complement activation. These observations are in accordance with the results in the present study, where the GG genotype is associated with risk for AMD and displays higher levels of complement activation than the CC genotype (Fig. 1A). Even though it plays such an important role, it was not part of the most predictive complotype in the present study.

Several *in vitro* studies have shown that having multiple SNPs in complement genes would lead to higher complement activation^20^,^35^. In the present study, the novel complotype composed of *CFB*(rs4151667)-*CFB*(rs641153)-*CFH*(rs800292) had a larger effect on complement activation than the initially studied complotype *C3* (rs2230199)-*CFB* (rs641153)-*CFH* (rs800292)^20^ (Table 1). The higher predictive value of the newly described complotype with respect to AMD might be related to the fact that it is composed of protective SNPs only rather than of a combination of polymorphisms with opposing effects on AMD susceptibility. When comparing the strongest effect (OR = 0.3) of this new complotype on the risk of AMD with the odds ratios of the 38 individual loci described in the newest AMD GWAS^36^, we notice that the effect size is close to both the *CFH* (OR = 0.38) and the *ARMS2* (OR = 2.81), albeit reverse, locus. It is worth mentioning that the OR of 0.3 for the complotype it is seen when comparing TA-GG-GA to TT-GG-GG which has only two alleles difference out of the six.

This study is the first to analyze this specific complotype combination for its association with AMD and complement activity. Although it would have been interesting to study the simultaneous presence of all four genotyped SNPs, cohorts even larger than ours are needed to avoid the problem of small genotype combination groups that cannot be reliably compared.

Intriguingly, the homozygous genotypes associated with the highest complement activation levels in all three SNPs (TT-GG-GG) in the novel complotype are found most frequently in both AMD patients and controls. This is in contrast to what was proposed in the theoretical model from^22^, where the extreme genotype combinations were expected to be at the lower end of the carrier frequency spectrum. With fewer than ten individuals for patients or controls, the combination of all heterozygous genotypes was rare. The combination where all SNPs had the homozygous protective genotypes (AA-AA-AA) was not present in our cohort at all. In our study, therefore, the frequency distribution is skewed towards complement-raising genotypes.

This could potentially be explained by the fact that our cohort has a mean age of 73 years and might be enriched, therefore, for alleles that promote survival. In this case, the alleles that give higher complement activation could offer better lifetime protection against infection. However, these same genetic variants would potentially induce low-grade inflammation, and its effect would only be observed later in life, as is the case for AMD, a disease that is prevalent in the elderly population. In support of this hypothesis, immune genes have been described to have the highest rate of positive selection^37^. Upon examination of the amino acid conservation of the SNPs in the present study, in humans three complement-raising variants are the refence amino acid, compared to only one in primates (Supplementary Table 4).

A significant difference in complement levels was observed between AMD patients and controls carrying the TT-GG-GA genotype combination. Although the highest mean difference was observed between the groups carrying the TT-GA-GG combination, this difference was not significant due to the high standard error.

The four most prevalent genotype combinations are all associated with high levels of complement activation in AMD patients with only minor differences between the groups. The three genotype combinations that are least prevalent are associated with lower complement activity. If we look at the specific genotype combinations, some interesting observations can be made.

First of all, the TT-GG-GG genotype combination is associated with the highest complement activation levels and is more prevalent in AMD patients (57.3%) than in controls (43.3%). The TA-GG-GG genotype, which is only different with respect to 1 risk allele in *CFB rs*4151667, is at the lower end of complement activation. The only other genotype combination with TA instead of TT for *CFB* rs4151667 is also associated with lower complement activation. This suggest that this SNP might be the most important of the three SNPs in the novel complotype and is the driving force behind the influence on complement activation. This is also evident in the results from the random forest analyses, where *CFB* rs4151667 is the strongest predictor for complement activation compared to the other individual SNPs.

Another interesting observation from Fig. 1 is the difference in complement activation between genotype combinations TT-GG-AA and TT-GA-GA. Both combinations include four risk alleles and two protective alleles, but the difference in complement activation is striking, especially in the AMD group. Perhaps the presence of two protective alleles in one SNP, as in the TT-GG-AA genotype combination, has a stronger influence on complement activity than the combination of two heterozygous SNPs (TT-GA-GA). Observations like ours may help to clarify this and warrant further research, preferentially in an even larger dataset.

One of the major strengths of this study is the use of the large EUGENDA dataset. To the best of our knowledge, this is one of the largest datasets of complement activation to date. For the evaluation of mean differences in complement activation at a population level, as we have done in this study, a single measurement of C3 and C3d in each individual is sufficient. However, if complement activation would be used on an individual basis, such as for the selection of patients for clinical trials, multiple measurements over time would be prefered to correct for individual variations in complement activation.

In conclusion, the current study has demonstrated that a novel complotype composed of *CFB* (rs4151667) in combination with *CFB* (rs641153) and *CFH*(rs800292) is strongly associated with complement activation and AMD status. These findings are relevant in the context of future complement-lowering treatments for AMD. In the era of personalized medicine, we are moving towards a more individualized approach to the treatment of diseases. To evaluate new treatment strategies, we need detailed information to determine how subgroups of patients with a higher treatment response potential should be defined. In this case, genotype-based patient stratification may identify those individuals that are genetically predisposed to having the highest complement levels, potentially making them the best candidates for complement-inhibiting therapies in AMD.

## Materials and Methods

### Study population

In this study, 3042 participants from the European Genetic Database (EUGENDA, www.eugenda.org), over the age of 50 years, were included. The study was performed in accordance with the tenets of the Declaration of Helsinki and the Medical Research Involving Human Subjects Act (WMO) and was approved by the local ethics committee of the University Hospitals in Cologne and Nijmegen. Written informed consent was obtained from all participants.

AMD and control status were assigned by multimodal image grading that included stereo fundus photographs, fluorescein angiograms and spectral domain optical coherence tomograms. The grading was performed according to the standard protocol of the Cologne Image Reading Center (CIRCL) by certified graders (TR, LE) as previously described^38^.

Age, gender and BMI were obtained by standardized interviewer-assisted questionnaires.

### Complement measurements and genetic analysis

Complement component C3 and the activation fragment C3d were measured in serum samples as previously described^9^, and the C3d/C3 ratio was calculated as a measure of complement activation. The complement activation data were skewed and had several outliers at the high end of the value range. In order to reduce the risk of outlier effects distorting the data, five percent of the highest values from the entire dataset were excluded from our analysis. After the exclusion of the outliers, the remaining skeweness of the C3d/C3 data was normalized by Log10 transformation.

Genomic DNA was extracted from peripheral blood samples using standard procedures. Four SNPs, *CFH* (rs800292), *CFB* (rs4151667), *CFB* (rs641153) and *C3* (rs2230199) were genotyped using the KASPar SNP Genotyping System by LGC Genomics.

### Statistical analysis

All associations were calculated using SPSS software version 20.0 (IBM Software and Systems, Armonk, NY, USA). Associations with complement activation were analyzed using General Linear Models with C3d/C3 as the dependent variable. The models were corrected for age, gender, BMI and disease status.

The associations between AMD phenotype and the individual SNPs or the complotype were evaluated using logistic regression. To determine if the SNPs were independently associated with the disease, all four SNPs were included in the logistic regression model at once.

To avoid being relevant only to our sample set (overfitting), the most informative complotype combination was determined by calculating the variable importance in a random forest analysis using the R package (RandomForest version 4.6-10). In the first analysis, C3d/C3 was included as the dependent variable for the regression type random forest test. In the second analysis, the disease status was defined as the classifier for a classification type of random forest. For both analyses the number of predictors sampled for splitting at each node was set to two. All other options were left at default setting.

## Additional Information

**How to cite this article**: Paun, C. C. *et al.* A Novel Complotype Combination Associates with Age-Related Macular Degeneration and High Complement Activation Levels *in vivo*. *Sci. Rep.* **6**, 26568; doi: 10.1038/srep26568 (2016).

## Supplementary Material

Supplementary Information

## Figures and Tables

**Table 1:** Variable importance scores of *C3*, *CFB* and *CFH* genotypes and genotype combinations on complement activation levels and AMD status.

Variables	%IncMSE	IncNode Purity	Mean Decrease Accuracy	Mean Decrease Gini
*C3* (rs2230199)	5.33	0.06	6.59	1.85
*CFB* (rs4151667)	13.24	0.20	2.51	0.92
*CFB* (rs641153)	5.77	0.11	6.08	2.41
*CFH* (rs800292)	6.07	0.15	13.24	6.57
*C3* (rs2230199)-*CFB* (rs4151667)-*CFB* (rs641153)	8.83	0.34	8.71	5.07
*C3* (rs2230199)-*CFB* (rs4151667)-*CFH* (rs800292)	9.08	0.35	13.12	9.66
*C3* (rs2230199)-*CFB* (rs641153)-*CFH* (rs800292)	10.88	0.33	13.84	11.85
*CFB* (rs4151667)-*CFB* (rs641153)-*CFH* (rs800292)	18.58	0.62	18.47	17.28
*C3* (rs2230199)-*CFB* (rs4151667)-*CFB* (rs641153)-*CFH* (rs800292)	10.25	0.49	14.07	15.36

Mean decrease accuracy and mean decrease Gini measure variable importance in predicting disease status. %IncMSE and IncNode Purity are measures for variable importance in predicting complement activation. For all variables, the highest values represent the best predictors.

**Table 2:** Association between the novel complotype and AMD.

Genotype combination for the novel complotype	N		P	OR	95% CI	
	Contols	AMD			Lower	Upper
TT-GG-GG	607	916	5.84*10^−13^	–	–	–
TA-GG-GA	47	23	1.01*10^−5^	0.3	0.174	0.51
TA-GG-GG	55	65	0.131	0.74	0.494	1.096
TT-GA-GA	74	47	6.65*10^−7^	0.36	0.237	0.535
TT-GA-GG	112	106	3.2*10^−4^	0.57	0.422	0.775
TT-GG-AA	59	48	0.007	0.56	0.373	0.856
TT-GG-GA	406	370	7.32*10^−9^	0.58	0.48	0.696

The model was established by logistic regression analysis, corrected for age and gender. Bonferroni corrected threshold for statistical significance is p < 0.008.
