# Supplementary material for: A Novel Complotype Combination Associates with Age-Related Macular Degeneration and High Complement Activation Levels in vivo
Source: Sci Rep. 2016 May 31;6:26568. doi: 10.1038/srep26568 (PMC4886525; doi:10.1038/srep26568)
Supplement: Supplementary Information [file srep26568-s1.pdf]

A Novel Complotype Combination Associates with Age-Related Macular Degeneration and High Complement Activation Levels *in vivo*

Constantin C. Paun[1,2], Yara T.E Lechanteur[1], Joannes M. M. Groenewoud[4], Lebriz Ersoy[3], Tina Schick[3], Mohamed R. Daha[5], Sascha Fauser[3], Carel B. Hoyng [1], Anneke I. den Hollander [1,2,6], Eiko K. de Jong* [1,2]

[1]Department of Ophthalmology, Radboud University Medical Center, Philips van Leydenlaan 15, 6525 EX, Nijmegen, The Netherlands

[2]Radboud Institute for Molecular Life Sciences, Radboud University Medical Center, Geert Grooteplein 28, 6525 GA Nijmegen, The Netherlands

[3]Department of Ophthalmology, University Hospital of Cologne, Kerpener Str. 62, 50924, Cologne, Germany

[4]Department for Health and Evidence, Radboud University Medical Center, Geert Grooteplein Noord 21, 6525 EZ, Nijmegen, The Netherlands

[5]Department of Nephrology, Leiden University Medical Center, Albinusdreef 2 2333 ZA, Leiden, The Netherlands

[6]Department of Human Genetics, Radboud University Medical Center, Geert Grooteplein 10, 6525 HB, Nijmegen, The Netherlands

Corresponding author: Eiko K de Jong,

Radboud University Medical Center, Department of Ophthalmology, internal zip: 409

Philips van Leydenlaan 15, 6525 EX, Nijmegen

The Netherlands

Eiko.deJong@radboudumc.nl

Supplementary Table 1. Association between AMD and SNP genotypes in the *CFH, CFB* and *C3* genes

SNP	cDNA change	Protein change	Genotype	N		P-value	OR	95% C.I.for EXP(B)	
				AMD	Control			Lower	Upper
CFH rs800292	c.134G>A	p.V62I	GG	1101	801	$7.4*10^{-11}$			
			GA	445	539	0.015	0.63	0.435	0.913
			AA*	62	77	$2.4*10^{-11}$	0.564	0.477	0.667
CFB rs4151667	c.26T>A	p.L9H	TT*	1511	1291	0.02			
			TA	99	127	0.904	0.828	0.038	17.888
			AA	1	1	0.005	0.654	0.486	0.881
CFB rs641153	c.95G>A	p.R32Q	GG*	1436	1195	$3*10^{-4}$			
			GA	171	217	0.387	0.545	0.138	2.152
			AA	4	7	$7.9*10^{-5}$	0.623	0.492	0.788
C3 rs2230199	c.304G>C	p.R102G	CC*	911	901	$2.4*10^{-6}$			
			CG	570	471	0.046	1.183	1.003	1.396
			GG	117	47	$7.8*10^{-7}$	2.59	1.775	3.777

Analyses were performed by logistic regression analysis. The genotypes marked with * are the ancestral variants. Variables entered in the model: *CFH* rs800292, *CFB* rs4151667, *CFB* rs641153, *C3* rs2230199, age and gender. Bonferroni corrected threshold for statistical significance is p<0.004.

Supplementary Table 2. Genotype combination frequency for the novel complotype

***CFB* (rs4151667) - *CFB* (rs641153) - *CFH* (rs800292)**	**Control**		**AMD**		**Total**
	**n**	**%**	**n**	**%**	
AA - GG - GG	1	0.1	1	0.1	2
TA - GA - AA	0	0.0	2	0.1	2
TT - AA – GA	1	0.1	1	0.1	2
TA - GA - GA	5	0.4	1	0.1	6
TT - AA - GG	6	0.4	3	0.2	9
TA - GG - AA	7	0.5	3	0.2	10
TA - GA - GG	10	0.7	5	0.3	15
TT - GA - AA	11	0.8	9	0.6	20
TA - GG - GA	47	3.4	23	1.4	70
TA - GG - GG	55	3.9	65	4.1	120
TT - GG - AA	59	4.2	48	3.0	107
TT - GA - GA	74	5.3	47	2.9	121
TT - GA - GG	112	8.0	106	6.6	218
TT - GG - GA	406	29.0	370	23.1	776
TT - GG - GG	607	43.3	916	57.3	1523
**Total**	1401	100	1600	100	3001

Supplementary Table 3. Differences in mean complement activation levels between genotype combinations

CFB (rs4151667) - *CFB* (rs641153) - *CFH* (rs800292)		Mean Difference (I-J)	Std. Error	P-value
TA - GG - GA	TA - GG - GG	-0.024	0.026	1
	TT - GA - GA	-0.041	0.027	1
	TT - GA - GG	-0.085*	0.024	0.011
	TT - GG - AA	-0.06	0.027	0.524
	TT - GG - GA	-0.079*	0.022	0.006
	TT - GG - GG	-0.100*	0.021	$6.1*10^{-5}$
TA - GG - GG	TA - GG - GA	0.024	0.026	1
	TT - GA - GA	-0.017	0.024	1
	TT - GA - GG	-0.061	0.021	0.074
	TT - GG - AA	-0.036	0.023	1
	TT - GG - GA	-0.055*	0.018	0.043
	TT - GG - GG	-0.076*	0.017	$1.9*10^{-4}$
TT - GA - GA	TA - GG - GA	0.041	0.027	1
	TA - GG - GG	0.017	0.024	1
	TT - GA - GG	-0.044	0.021	0.843
	TT - GG - AA	-0.019	0.024	1
	TT - GG - GA	-0.038	0.018	0.826
	TT - GG - GG	-0.059*	0.018	0.019
TT - GA - GG	TA - GG - GA	0.085*	0.024	0.011
	TA - GG - GG	0.061	0.021	0.074
	TT - GA - GA	0.044	0.021	0.843
	TT - GG - AA	0.025	0.021	1
	TT - GG - GA	0.006	0.014	1
	TT - GG - GG	-0.015	0.014	1
TT - GG - AA	TA - GG - GA	0.06	0.027	0.524
	TA - GG - GG	0.036	0.023	1
	TT - GA - GA	0.019	0.024	1
	TT - GA - GG	-0.025	0.021	1
	TT - GG - GA	-0.019	0.018	1
	TT - GG - GG	-0.04	0.018	0.464
TT - GG - GA	TA - GG - GA	0.079*	0.022	0.006
	TA - GG - GG	0.055*	0.018	0.043
	TT - GA - GA	0.038	0.018	0.826
	TT - GA - GG	-0.006	0.014	1
	TT - GG - AA	0.019	0.018	1

	TT - GG - GG	-0.021	0.008	0.215
TT - GG - GG	TA - GG - GA	0.100*	0.021	$6.1*10^{-5}$
	TA - GG - GG	0.076*	0.017	$1.9*10^{-4}$
	TT - GA - GA	0.059*	0.018	0.0189
	TT - GA - GG	0.015	0.014	1
	TT - GG - AA	0.04	0.018	0.464
	TT - GG - GA	0.021	0.008	0.215

*The mean difference is significant at the 0.05 level. All p-values were adjusted for multiple comparisons: Bonferroni. The general linear model was corrected for age, gender, BMI and disease status.

Supplementary Table 4. Amino acid conservation for *CFH* (rs800292, p.V62I) - *CFB* (rs4151667, p.L9H) - *CFB* (rs641153, p.R32Q) - *C3* (rs2230199, p.R102G)

Species	CFH p.V62I	CFB p.L9H	CFB p.R32Q	C3 p.R102G
**Human**	V	L	R	R
**Chimp**	I	L	Q	R
**Mouse**	I	L	R	G
**Dog**	I	L	A	G
**Cat**		L	G	G
**Caw**		L	G	G

Statistical Syntax used for the models built in SPSS and the R script used to run the Random forest analyses:

SPSS syntax for the statistical models:

UNIANOVA Log_C3d_C3 BY Gender Disease_status Complotype_SNP2_SNP3_SNP4 WITH Age_Blooddate Q14_BMI

/METHOD=SSTYPE(3)

/INTERCEPT=INCLUDE

/EMMEANS=TABLES(Complotype_SNP2_SNP3_SNP4) WITH(Age_Blooddate=MEAN Q14_BMI=MEAN) COMPARE

ADJ(BONFERRONI)

```
 /PRINT=ETASQ PARAMETER
 /CRITERIA=ALPHA(.05)
 /DESIGN=Gender Disease_status Complotype_SNP2_SNP3_SNP4 Age_Blooddate Q14_BMI.

LOGISTIC REGRESSION VARIABLES Disease_status
 /METHOD=ENTER Complotype_SNP2_SNP3_SNP4 Age_Blooddate Gender
 /CONTRAST (Complotype_SNP2_SNP3_SNP4)=Indicator
 /CLASSPLOT
 /PRINT=CI(95)
 /CRITERIA=PIN(0.05) POUT(0.10) ITERATE(20) CUT(0.5).
```

The R script used for the random forest analyses

```
library(randomForest)
setwd("")
data <- read.table("file.txt", header=T)
View(data)
Combi3_4_SNPs <- data[,(2:11)]
attach(Combi3_4_SNPs)
set.seed(4)
complotype.rf <- randomForest(Log_C3d_C3 ~ ., data=Combi3_4_SNPs, mtry=2, importance=TRUE)
print(complotype.rf)
round(importance(complotype.rf), 2)

library(randomForest)
setwd("")
```

```
data <- read.table("file2.txt", header=T)
View(data)
selected_colums <- data[, (2:11)]
names(selected_colums)
attach(selected_colums)
sink("results_randomeForest_on_disease_status.txt")
set.seed(4)
complotype_on_AMD.rf <- randomForest(Disease_status ~ ., data=selected_colums,
importance=TRUE, proximity=TRUE)
print(complotype_on_AMD.rf)
round(importance(complotype_on_AMD.rf), 2)
```
